# Paired-Agent Fluorescence Molecular Imaging of Sentinel Lymph Nodes Using Indocyanine Green as a Control Agent for Antibody-Based Targeted Agents

**DOI:** 10.1155/2019/7561862

**Published:** 2019-01-03

**Authors:** Chengyue Li, Xiaochun Xu, Nathan McMahon, Omar Alhaj Ibrahim, Husain A. Sattar, Kenneth M. Tichauer

**Affiliations:** ^1^Biomedical Engineering, Illinois Institute of Technology, Chicago, IL 60616, USA; ^2^Department of Pathology, University of Chicago, Chicago, IL 60637, USA

## Abstract

**Purpose:**

Paired-agent molecular imaging methods, which employ coadministration of an untargeted, “control” imaging agent with a targeted agent to correct for nonspecific uptake, have been demonstrated to detect 200 cancer cells in a mouse model of metastatic breast cancer. This study demonstrates that indocyanine green (ICG), which is approved for human use, is an ideal control agent for future paired-agent studies to facilitate eventual clinical translation.

**Methods:**

The kinetics of ICG were compared with a known ideal control imaging agent, IRDye-700DX-labeled antibody in both healthy and metastatic rat popliteal lymph nodes after coadministration, intradermally in the footpad.

**Results:**

The kinetics of ICG and antibody-based imaging agent in tumor-free rat lymph nodes demonstrated a strong correlation with each other (*r* = 0.98, *p* < 0.001) with a measured binding potential of −0.102 ± 0.03 at 20 min postagent injection, while the kinetics of ICG and targeted imaging agent shows significant separation in the metastatic lymph nodes.

**Conclusion:**

This study indicated a potential for microscopic sensitivity to cancer spread in sentinel lymph nodes using ICG as a control agent for antibody-based molecular imaging assays.

## 1. Introduction

Identification of cancer spread to tumor draining (sentinel) lymph nodes through lymph node dissection and histology assessment is an established staging procedure in the management of many cancers, predominantly breast carcinoma and melanoma [1]. The clinical protocol in breast carcinoma varies slightly from institute to institute but typically involves (1) node localization through lymphoscintigraphy (gamma probe detection of ^99m^Tc-sulfur colloid spread from peritumoral or subareolar injection site to tumor-draining lymph node(s) [2]) and/or visualized blue dye mapping [3], (2) surgical dissection of the lymph node, and (3) histological examination by a trained pathologist who manually scans select slices of the H&E stained lymph node for cancer cell presence [4]. However, the procedure is associated with overtreatment concerns and some considerable morbidity, including lymphedema, seroma formation, numbness, and restricted arm movement [5, 6]. Furthermore, histology analysis of lymph nodes can be time-consuming and delay subsequent procedures; and pressure to achieve earlier detection of more aggressive breast cancer has led to an increasing number of dissections on tumor-free nodes: estimated to be higher than 70% [7]. Motivated by these drawbacks, considerable efforts have been made to develop imaging techniques that can assess the metastatic status of sentinel lymph nodes noninvasively [8].

The more promising imaging methods have aimed at quantifying tumor burden through accumulation of cancer-targeted imaging agents [9–13] or through aberrations in the transport kinetics of untargeted imaging agents [14, 15]. With respect to targeted imaging agent studies, a complication is that variable delivery and nonspecific uptake/retention of the imaging agents in lymph nodes can significantly confound sensitivity and specificity. A novel approach, paired-agent imaging of the sentinel lymph node (PAISLY), was developed to account for the variable delivery and nonspecific uptake/retention of cancer-targeted imaging agents by coadministering an untargeted, “control” imaging agent. Recently, in a metastatic mouse model, PAISLY was demonstrated to be capable of quantitatively estimating the number of cancer cells in sentinel lymph nodes with a sensitivity of fewer than 200 cells [16], which matches or may exceed the sensitivity of invasive standard lymph node biopsy.

While these early results using PAISLY are encouraging, the approach's requirement that two different imaging agents be injected could represent a substantial barrier towards clinical adaptation of this methodology. One way to accelerate the clinical translation of PAISLY is to utilize imaging agents that have already been approved by the Food and Drug Administration (FDA) for human use. At present, all FDA-approved fluorescent imaging agents are untargeted—*i.e.*, they do not bind specifically to cancer—and could therefore be suitable to be used as control agents in PAISLY. The FDA-approved indocyanine green (ICG), in particular, has several characteristics that make it a promising control imaging agent candidate. Specifically, its fluorescence emission spectrum is in the near-infrared where light absorption by tissue is minimal, it exhibits low toxicity and rapid excretion [17, 18], it has been used off-label for localization of lymph nodes [19–24], and its effective size (after binding to plasma proteins) is similar to the most commonly used targeted imaging agents, supporting the potential for similar kinetics in the lymphatic system [25].

A number of studies have demonstrated the potential for fluorescently labeled cancer targeted antibodies to detect different forms of cancer in the operative setting both preclinically and clinically [26–30]. Sensitivity and specificity of cancer detection using these antibodies could be improved by employing PAISLY protocols, and recently, fluorescently labeled nontargeted immunoglobulin G (IgG) antibody has been shown to be an ideal control agent for these antibody-based targeted agents [16]. However, fluorescently labeled IgG is not FDA-approved. The purpose of the work presented in this paper was to demonstrate that ICG, an FDA-approved dye, could be used to replace fluorescent IgG for antibody-based PAISLY studies by comparing the kinetics of ICG and fluorescently labeled antibody in rat lymph nodes. In brief, it was found that the protocol of imaging agent mixing and concentration of ICG affected the similarity in the kinetics and an optimal strategy is presented.

## 2. Materials and Methods

### 2.1. Paired-Agent Imaging of Sentinel Lymph Node (PAISLY)

**Figure 1:**
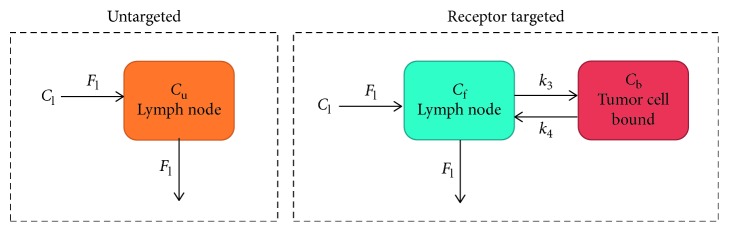
Compartment model for targeted and control agent kinetics in a lymph node. In each case, the agents are assumed to enter and leave any lymph node region-of-interest at a rate of the lymph fluid flow (*F*_l_), governed by the concentration input curve (*C*_l_). The targeted agent is then able to reach an equilibrium between bound (*C*_b_) and unbound (*C*_f_) states with a rate constant of binding, *k*_3_, and a rate constant of dissociation, *k*_4_. The control agent is unable to bind and is only assumed to be in a freely associated space, *C*_f,*T*_.

The paired-agent imaging of the sentinel lymph node (PAISLY) approach was derived from “paired-agent” compartmental modelling where the kinetics of a control imaging agent is used to account for nonbinding related kinetics of a cancer-targeted imaging agent (Figure 1) [31]. By coinjecting the targeted and control agents and concurrently monitoring their uptake and retention in a tumor-draining lymph node, it is possible to account for delivery variability and nonspecific retention of the targeted agent, enabling the concentration of targeted receptors to be quantified through kinetic modelling. Specifically, the compartment model describes the concentration of the targeted imaging agent as a function of time, *t*, in a lymph node as a sum of two concentrations: unbound, *C*_f_(*t*), and bound, *C*_b_(*t*). The concentration of the agent in the upstream, afferent lymphatic vessels, *C*_l_(*t*), enters and drains the lymph node at a lymphatic flow rate, *F*_l_. Rate constants *k*_3_ and *k*_4_ describe targeted agent-to-receptor association and dissociation, respectively. The compartment model for the control agent is identical to the targeted with an absence of a bound compartment, and *C*_f_ is relabeled *C*_f,C_ (control agent-free concentration) to represent a possible discordance in the amount of freely associated targeted and control agent owing to the influence of binding. The ratio of *k*_3_/*k*_4,_ known as the “binding potential” (BP) [32] is the parameter of interest from the compartment model as it is equivalent to the product of the concentration of the targeted receptor and the affinity of the targeted agent for the receptor (the latter of which can be measured *ex vivo*) [33]. If the control agent concentration measured in the lymph node is similar to the unbound concentration of the targeted agent (*i.e*., if *C*_f,C_ ≈ *C*_f_), then the difference of the targeted agent concentration (*C*_f_  + *C*_b_) and the control agent concentration (≈*C*_f_) will be approximately equal to *C*_b_. Dividing by the control agent concentration results in *C*_b_/*C*_f_, which can be shown to be equivalent to *k*_3_/*k*_4_ by assuming the adiabatic approximation (free and bound compartment always in equilibrium) [34], the following equation describes the rate of change in time of the bound-targeted agent concentration:

(1) $$\begin{array}{c} \frac{dC_{\text{b}}}{dt}=k_{3}C_{\text{f}}-k_{3}C_{\text{b}}. \end{array}$$

Therefore, the binding potential can be estimated by the following:

(2) $$\begin{array}{c} \text{BP}=\frac{\text{Tar}-\text{Contr}}{\text{Contr}}, \end{array}$$

where Tar is the measured concentration of the targeted agent and Contr is the measured concentration of the control agent in the lymph node. Much of this work stems from previous publications that have demonstrated the development and validation of this approach for intravascular coadministration of targeted and control agents [31].

### 2.2. ICG Preparation

Indocyanine green (ICG; Chem-Impex International, Inc., Wood Dale, IL) was dissolved in dimethyl sulfoxide (DMSO, Amresco, Solon, OH) to yield a 1 M concentration stock solution for repeated use due to the instability of ICG in aqueous solution [35]. To prepare single doses, the ICG stock solution was diluted with phosphate-buffered saline (PBS) to obtain a series of solutions with a range of final concentrations. ICG molecules are known to bind rapidly and almost completely to plasma proteins [36], which increases fluorescence efficiency and thus detection sensitivity [37]. To study ICG characteristics in blood plasma *in vitro*, bovine serum albumin (BSA; Sigma-Aldrich, St. Louis, MO) was chosen as a model for ICG conjugation, as a result albumin is the main protein of blood plasma [38]. A solution was created by dissolving 40 mg of BSA in 1 mL of distilled water. For *in vivo* fluorescence imaging, rat albumin (MP Biomedicals, Santa Ana, CA) was used to compose an ICG : albumin complex before use of injection.

### 2.3. Synthesis of Antibody-Based Imaging Agent

To test the ability for ICG to play a role as the control imaging agent, the delivery and retention of fluorescent antibody imaging agents and ICG in lymphatics were compared. Rat immunoglobin G (IgG) (MP Biomedicals, Santa Ana, CA), a nonspecific antibody to mimic antibody-based imaging agent in the absence of binding, and cetuximab (provided by Davis Lab at Dartmouth College), an EGFR-specific antibody that selected to act as a targeted imaging agent in the metastatic lymph node model, were both evaluated against ICG. Both antibodies were labeled with the NHS ester form of a fluorescent dye, IRDye-700DX (LICOR Biosciences, Lincoln, NE) using manufacturer-supported protocols. In brief, 2 mg of antibody was dissolved in 200 *μ*L of PBS. 20 *μ*L of 1M sodium bicarbonate solution was then added to the solution to increase pH for optimal binding to the fluorophore as per manufacturer instructions. The fluorophore was dissolved in DMSO and added to the antibody solution in a 5 : 1 dye-to-antibody ratio. Note: DMSO, while not used in the clinical formulation of ICG, was used here to help dissolve low quantities of ICG powder for animal use and improve stability of ICG in the aqueous solution as per manufacturer recommendations. End concentration of DMSO was less than 0.01% v/v, so we do not expect this to change overall findings compared to the clinical formulation. The final solution was covered with an aluminum foil to protect it from light and then mixed on a magnetic stir plate for 2 h at room temperature. The fluorophore-labeled antibody conjugate was separated from free dye by using a 40 K Zeba spin desalting column (Thermo Fisher Scientific). The final concentration of antibody-based imaging agent was measured by a NanoDrop 2000 spectrophotometer (Thermo Fisher Scientific).

### 2.4. ICG Characteristics in Albumin *In Vitro*

The ICG fluorescence signals from 800–840 nm were measured for a range of ICG concentrations (0.001 to 1000 *μ*M) when mixed with BSA. A concentration of BSA equivalent to albumin concentrations observed in the lymphatic system [39] was used to create the same binding-site condition. ICG concentrations within a linear signal range (i.e., a concentration range where the fluorescence and concentration of ICG displayed a linear relationship) were identified to determine appropriate dosing levels of ICG concentration for the *in vivo* study.

The influence of protein binding on the ICG fluorescence quantum yield was determined for mixtures of ICG concentrations (between 0.1 and 25 *μ*M in PBS) and BSA concentrations (between 0 and 75 *μ*M in distilled water). All solutions were protected from light and tested immediately after preparation. 200 *μ*L of each sample was added to a separate well in a flat bottom 96-well plate. Fluorescence intensity of ICG was evaluated for investigating the binding properties of albumin with ICG on a wide-field fluorescence scanner (Pearl® Imager, LI-COR Biosciences).

The influence of preloading time of ICG and albumin was determined at an ICG concentration of 1 *μ*M and an albumin concentration of 50 *μ*M. 10 mL total of ICG and albumin were gently mixed, and then 200 *μ*L samples for each well were taken from the mixture and placed into a well in a flat bottom 96-well plate at the first 1 min, followed by immediate imaging on the Pearl; then, 6 time points were recorded at 5 min interval up to a maximum of 30 min. Tested samples were discarded once exposed to light to prevent effects of photobleaching. Sample mixtures were covered with an aluminum foil to prevent any exposure to light at room temperature, during the mixing.

Since ICG signal and lymph kinetics were optimal when prebound to albumin prior to injection and the ICG needed to be mixed with the fluorescently labeled antibody for coadministration, the effect of the mixing order of the imaging agents and albumin was also evaluated at concentrations of 1 *μ*M ICG, 0.2 *μ*M fluorescent antibody, and 50 *μ*M albumin (determined to be optimal based on the initial experiments described in this paper). In the preparation of the first mixing group, ICG and albumin were allowed to mix in a light-protected container for 15 min at room temperature. Subsequently, the antibody-based imaging agent was added to the mixture and gently mixed. 200 *μ*L samples were placed in a flat bottom 96-well plate, and ICG and IRDye-700DX-antibody fluorescence intensities were recorded immediately after sample preparation on the Pearl Imager. The second mixing group was prepared by adding the antibody-based imaging agent and albumin at the beginning, mixed for 15 min, and then ICG was added. In the third mixing group, ICG and antibody-based imaging agent concentrations were added together before mixing with albumin. Except for the order of addition, the same mixing and imaging protocols were used as mentioned above for the first group. ICG fluorescence of each group was then compared to determine the suitable mixing order for solution mixture.

### 2.5. Animals

All animal experiments were carried out in accordance with the Institutional Animal Care and Use Committee (IACUC) at Illinois Institute of Technology under an approved protocol. All rats were fed a fluorescence-free diet (Teklad global 16% protein rodent diet, 2016S, Envigo) for a week prior to imaging to reduce tissue autofluorescence. In order to reduce autofluorescence from fur, animals were shaved and a commercial topical hair removal cream (Nair®) was applied over hind legs 24 h prior imaging. Only one hind foot was tested and injected per animal to image uptake in a model lymph node system: the popliteal lymph node. During imaging, breathing motion effects were minimized by lightly applying tape to the extended leg that was imaged. We do not expect the tape to alter lymph flow appreciably based on the similarity of lymph dynamics observed when the toes were taped instead of the leg (results not shown).

### 2.6. Control Lymph Node Model

Female Fischer 344 rats (*n* = 5) with an average weight of 245 ± 12 g (Envigo, Indianapolis, IN) were used as the normal lymph node model. To test the feasibility of ICG as the control imaging agent, the lymph node delivery and retention of an antibody-based imaging agent (Rat IgG-labeled IRDye-700DX) was first chosen to compare kinetics with ICG in the absence of binding.

### 2.7. Metastasis Lymph Node Model

**Figure 2:**
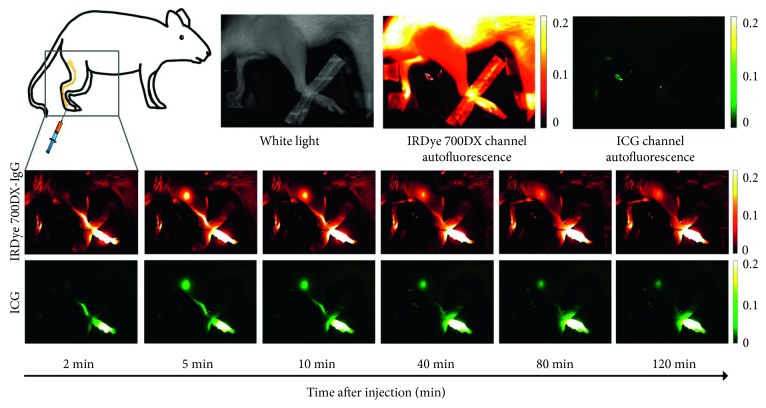
Images of a single rat after paired-agent injection of an antibody-based imaging agent and ICG in the rear footpad are shown. Preinjection images were acquired to evaluate the background levels caused by autofluorescence. The time series images show the uptake and retention of both imaging agents. The top row represents the time-course signal acquired at the 700 nm channel (false-colored red), indicating antibody-imaging agent fluorescence, and the bottom row represents the signal acquired at the 800 nm channel (false-colored green), indicating ICG fluorescence.

To evaluate the potential using ICG as the control agent to detect tumor burden in tumor-draining lymph nodes, female athymic nude rats (*n* = 4) with an average weight of 179 ± 5 g (Hsd:Foxn1^rnu^, Envigo, Indianapolis, IN) were used in the experiment. 1 × 10^6^ green fluorescent protein-transfected human breast cancer cells MDA-MB-231-D3H2LN-GFP, known to overexpressed epidermal growth factor receptor (EGFR), were implanted into the right hind footpad. After 5 weeks, allowing tumors to grow and metastasize to the lymph node after implantation, rats were imaged (see Figure 2) with IRDye700-DX-labeled cetuximab as the targeted agent and ICG as the control agent, and popliteal lymph nodes were dissected upon euthanization. The presence of cancer cells MDA-MB-231-D3H2LN-GFP in the dissected lymph node was frozen and serial sectioned on a cryostat microtome (Shandon Cryotome E, Thermo Electron Corp., Marietta, OH) at 100 *μ*m intervals and validated by a fluorescence microscope (Zeiss, Thornwood, NY).

### 2.8. *In Vivo* Fluorescence Imaging

Each rat was anesthetized with 1.5–3% isoflurane (Butler Schein Animal Health, Dublin, OH) in 1.5 L/min oxygen (level of anesthesia was monitored by response to toe-pinch). The animals were then placed in a lateral position on the heated bed (37°C) of a Pearl Impulse Small Animal Imaging System (LICOR Bioscience, Lincoln, NE) with the right hind leg lightly taped out on a nonfluorescence box to expose the popliteal lymph node behind the knee and reduce unwanted motion from animal breathing. A 50 *μ*L volume of imaging agent mixture for each animal was prepared prior imaging. 0.05 nanomoles of ICG in PBS were preloaded in 50 *μ*M of rat albumin for 15 min before adding 0.01 nanomoles of the antibody-based imaging agent. A preinjection image was acquired to evaluate the background levels caused by autofluorescence. Subsequently, the cocktail of two imaging agents were then coinjected intradermally using a 30-gauge syringe-needle (BD PrecisionGlide Needle) into the right rear footpad of rats. Caution had to be taken while injecting to ensure the needle was not too deep as deep injections lead to delayed uptake of imaging agents into the lymphatic system (results not shown). Immediately after injection, white light and fluorescence at 700–740 nm and 800–840 nm (from 685 and 785 nm excitation, respectively) were acquired at 1-minute intervals for 2 h to obtain the uptake and retention dynamics of both imaging agents in the popliteal lymph nodes. Temporal images were then analyzed using MATLAB (R2015b, Mathwork, Natick, MA) on a pixel-by-pixel basis. A circular region-of-interest of 3.4 mm diameter was selected over the visible lymph node, a pixel-based normalization of tracer inputs (control signal was normalized to the targeted signal) immediately after injection was performed as previously reported by Kanick et al. [40], and the time course of the binding potential (BP) was produced for further analysis based on equation (2).

### 2.9. Statistical Analyses

SPSS 23 (IBM) was used for all statistical analyses. A repeated-measures ANOVA, with time as a within-subjects variable and imaging agent as a between-subjects variable, was used to identify the presence of any statistically significant differences between the temporal kinetics of IRDye-700DX-IgG and ICG in the rat popliteal lymph nodes imaged. Paired *t*-tests with a Bonferroni correction to correct for multiple comparisons were used to identify statistically significant differences in signals at different time points. A linear regression was also used to evaluate the strength of correlation between the ICG and IRDye-700DX-IgG signals over time, reporting Pearson's correlation coefficient. Statistical significance was based on *p* < 0.05. All data are presented as mean ± SD unless stated otherwise.

## 3. Results

### 3.1. *In Vivo* Fluorescence Imaging

#### 3.1.1. Control Lymph Node Model

As a first *in vivo* step in demonstrating the feasibility of using ICG as a control imaging agent, the optimized combined solution of albumin, ICG, and fluorescent antibody (*in vitro* results below) was injected into the footpads of nontumor bearing, control rats (*n* = 5 with IRDye 700DX-IgG + ICG + albumin; *n* = 1 with IRDye 700DX-cetuximab + ICG + albumin). The goal was to demonstrate the similarity in the lymph node kinetics of ICG and two types of fluorescent antibodies. Figure 2 presents representative fluorescent images of a rat's hind leg at the 700 nm channel in the Pearl System (shown in red) and the 800 nm channel in the Pearl System (shown in green), and preinjection images and a series of fluorescent images were taken over time after a pair-agent injection in a typical control animal.

**Figure 3:**
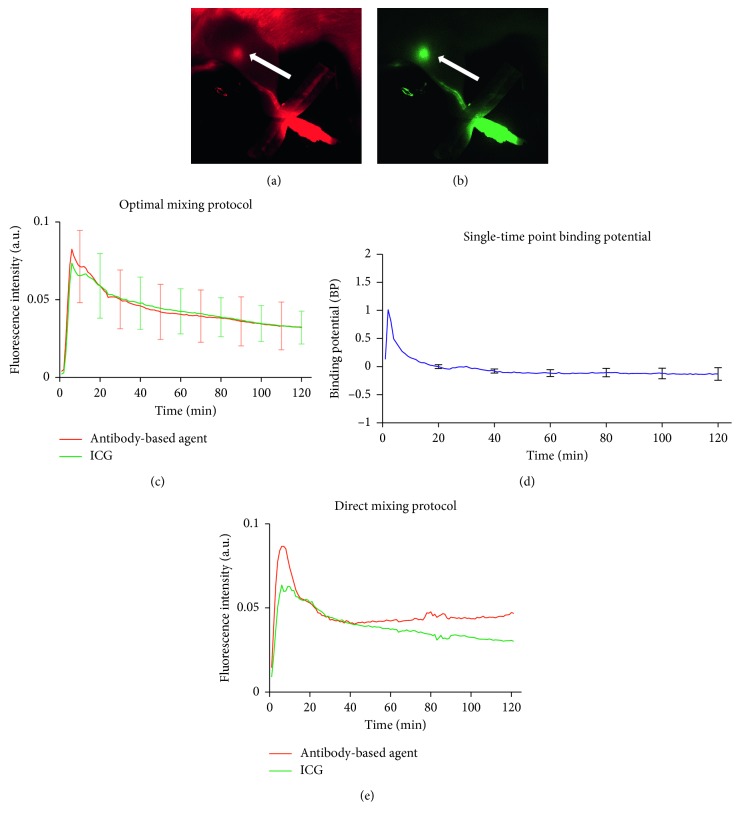
Kinetics of the antibody-based agent (IRDye-700DX-IgG) and ICG signal in a rat popliteal lymph node over time after intradermal footpad injection. Fluorescence images of ICG (green) and antibody-based agent (labeled IRDye-700CW-IgG; red) in the popliteal lymph node at 1 h after injection of the right rear footpad are presented in (a) and (b), respectively. The time course of fluorescence from both imaging agents failed to correlate with each other when all agents were mixed simultaneously just prior to injection as shown in (e). After the correction of mixing order and the preloading time, the potential ability for ICG to mimic the delivery and retention of an antibody-based imaging agent is presented in (c), depicting the mean fluorescence for both imaging agents in *n* = 5 rats in the lymph nodes as a function of time (error bars are SE). The binding potential for lymph nodes as a function of time after injection is presented in (d), with a maximum error of less than 0.05 in the absence of binding, further offering strong support that ICG is ideal for use as a control imaging agent in the PAISLY approach.

Figure 3 presents an overview of the paired-agent imaging of the sentinel lymph node (PAISLY) methods using ICG as a control agent to compare with an antibody-based imaging agent in the absence of binding. Figures 3(a) and 3(b) display the uptake of the antibody-based imaging agents (IRDye 700DX-IgG and IRDye 700DX-Cetuximab) and the uptake of ICG, from an injection site in the rear footpad of a rat to the popliteal lymph node 1 h after injection. A background subtraction algorithm was applied to both imaging agent uptake curves to minimize the interference from autofluorescence (subtraction of preinjection fluorescent images from all postinjection images; note: typical preinjection images are presented in Figure 2). In all the rat lymph nodes, the temporal uptake of ICG and antibody-based imaging agent matched each other within a 5% difference at all time points by 20 min after the injection. The measured time course of fluorescence from both ICG and IRDye-700DX-IgG in the popliteal lymph nodes (*n* = 5) for 2 h is shown in Figure 3(c). The results show the kinetics of the ICG and antibody-based imaging agent demonstrated a strong correlation with each other over 2h imaging (*r* = 0.977, *p* < 0.001), and no statistically significant differences were observed in the time-by-imaging agent or imaging agent omnibus tests in the repeated-measures ANOVA. The measured binding potential of IRDye-700DX-IgG measured by equation (2) is presented as a function time after injection in Figure 3(d). On average, the measured BP was −0.102 ± 0.03 after 20 min injection and did not change significantly over time as determined by the repeated-measures ANOVA. However, these data were obtained only under certain preloading time of ICG and albumin. Otherwise, the signal of both imaging agents failed to correlate consistently with each other by simply mixing the IRDye-700DX-IgG with the ICG and albumin at the same time as shown in Figure 3(e) (*n* = 1 demonstration without statistics).

#### 3.1.2. Metastasis Lymph Node Model

**Figure 4:**
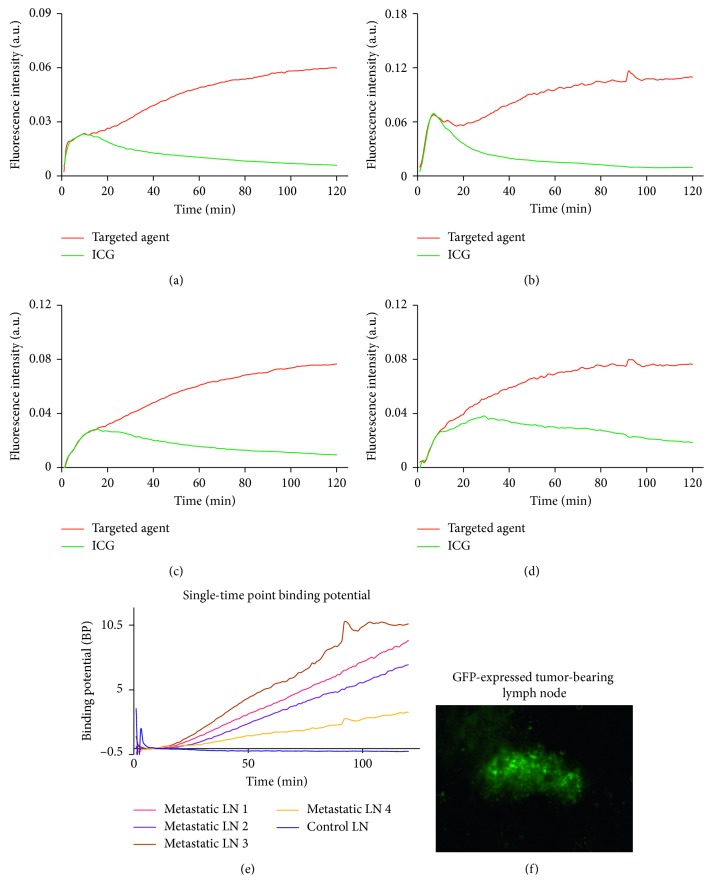
Kinetics of the antibody-based targeted agent (IRDye-700DX-cetuximab) and ICG signal in rats metastatic popliteal lymph nodes over time after intradermal footpad injection. The potential of using ICG as the control agent to detect metastases in *n* = 4 rats tumor-draining lymph nodes are presented in (a)–(d). The binding potential as a function of time for various lymph nodes after injection is presented in (e), including the binding potential of a control node. The presence of cancer in all metastatic lymph nodes was validated based on the presence of green fluorescent protein (GFP) fluorescence signal in microscopy since the cells were transfected with the gene for GFP, for example, which is presented in (f).

As a second *in vivo* step in demonstrating the feasibility of using ICG as a control imaging agent, the optimized combined solution of albumin, ICG, and fluorescent antibody (see *in vitro* results below) was injected into the footpads of tumor-bearing rats (*n* = 4 with IRDye 700DX-cetuximab + ICG + albumin). The goal was to demonstrate enhanced retention of the targeted antibody compared to ICG in the presence of cancer.  Figure 4 presents an overview of paired-agent imaging of the sentinel lymph node (PAISLY) methods using ICG as a control agent to detect metastases in tumor-draining lymph nodes. The measured time-course fluorescence from both ICG and IRDye 700DX-cetuximab in metastatic popliteal lymph nodes (*n* = 4) for 2 h is shown in Figures 4(a)–4(d). The results demonstrate the enhanced retention of the targeted imaging agent compared to ICG in all lymph nodes.  Figure 4(e) summaries the kinetics curves of binding potentials of all four metastatic popliteal lymph nodes as well as a cancer-free lymph node as control. Binding potential of metastatic nodes all increased over time while that of the control nodes remained constant near zero. In addition, the presence of GFP-expressing cancer cells in all metastatic lymph nodes were verified with fluorescence images of lymph node sections. A representative image of a lymph node section containing GFP-expressing cancer cells is shown in Figure 4(f).

### 3.2. *In Vitro* Fluorescence Imaging

**Figure 5:**
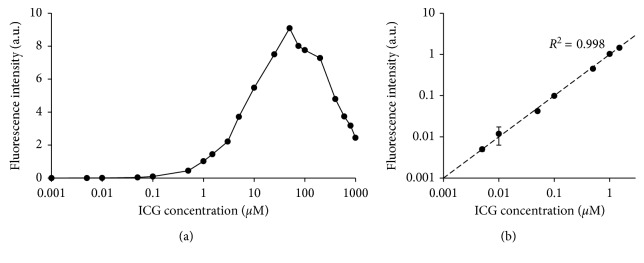
Fluorescence intensity of ICG in albumin as a function of ICG concentration. Fluorescence maximum was observed at 50 *μ*M (quenching threshold) with the concentration range 0.001–1000 *μ*M in albumin concentration equivalent to concentration in the lymphatic system (a). A correlation between ICG concentration and its fluorescence intensity is presented in (b). The dashed line represents the linear regression of the data.

With the known property of ICG that it sticks to proteins, a number of experiments were carried out to determine the optimal way to combine ICG with antibody-based fluorescent-imaging agents for paired-agent imaging.  Figure 5 displays the fluorescence intensity of ICG in an albumin concentration equivalent to that observed in the lymphatic system (21 g/L) as a function of ICG concentration. The ICG concentration was ranged from 0.001 *μ*M to 1000 *μ*M. The plot demonstrates that fluorescence intensity of ICG increased with concentration up to an ICG concentration of 50 *μ*M. Higher concentrations of ICG exhibited nonlinearity in the fluorescence signal with concentration and gradually decreased with an increase in concentration (Figure 5(a)). This result indicated that ICG self-quenching in albumin is obtained at concentrations of 50 *μ*M and above. In order to avoid self-quenching effects, care must be taken to ensure that the ICG concentration used in the experiment is far below the quenching threshold. To determine the optimal working concentration of ICG, the correlation of concentration and fluorescence intensity of ICG within a linear range was identified. The relationship of ICG concentration within 0.005–1.5 *μ*M and its fluorescence intensity was highly significant (*r* = 0.999, *p* < 0.001; Figure 5(b)).

**Figure 6:**
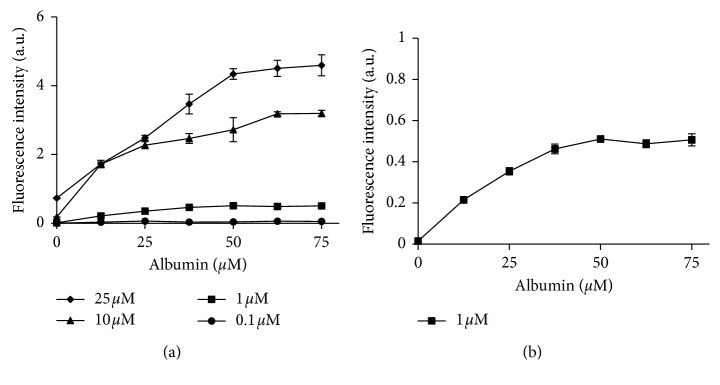
Fluorescence intensity of ICG as a function of albumin concentration with various ICG concentrations. Higher concentrations of ICG required higher concentration of albumin to reach a maximum fluorescence intensity (a). ICG fluorescence plateaued at 50 *μ*M albumin with 1 *μ*M ICG suggesting that it reached its maximum with all ICG bound to albumin in a ratio of 50 : 1 (albumin : ICG) (b).

Figure 6 shows the plot of fluorescence intensity of ICG versus albumin concentration. The ICG concentration was tested over a range from 0.1 *μ*M to 25 *μ*M, along with the albumin concentration from 0–75 *μ*M. The plot demonstrates a substantial increase in ICG fluorescence intensity upon albumin binding. As the concentration of albumin was increased, the fluorescence intensity of ICG also increased until the signal reached a plateau. The concentration of albumin for ICG to reach to its maximum fluorescence was dependent on the ICG concentration used. As the concentration of ICG was increased, it required higher concentrations of albumin to saturate ICG binding.  Figure 6(b) demonstrates that, at an ICG concentration of 1 *μ*M, fluorescence intensity asymptotes to a maximum at an albumin concentration of approximately 50 *μ*M, after which there is no further increase in the ICG signal despite the concentration of albumin increase. This result suggests that a 50 *μ*M albumin concentration is sufficient for all ICG (at 1 *μ*M) to reach an ICG-albumin binding maximum.

**Figure 7:**
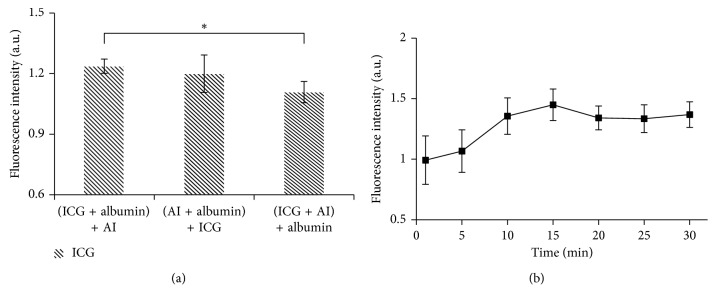
Influence of the mixing order and preloading time of ICG and albumin on ICG fluorescence. Fluorescence from ICG varied when mixing in different orders of ICG, albumin, and antibody-based agent (AI) IRDye-700DX-IgG as presented in (a). ICG preloaded with albumin prior to mixing with the antibody-based agent allowed ICG to reach its highest intensity without quenching its fluorescence. The fluorescence profile of ICG in albumin as a function of time is shown in (b). Fluorescence intensity of ICG was maximum at 15 min, suggesting that ICG has completely bound to albumin at this point.

Figure 7(a) shows the effect of the mixing order of imaging agents on ICG fluorescence intensity. The concentrations used were 1 *μ*M, 0.2 *μ*M, and 50 *μ*M for ICG, antibody-based imaging agent, and albumin, respectively. ICG fluorescence was observed to be highest in the solutions where ICG and albumin were premixed prior to the addition of the antibody agent and lowest when ICG was premixed with the antibody. The premixing of antibody and albumin prior to addition of ICG lead to a signal somewhere between what was observed in the other two mixing protocols. The results showed statistically significant differences between the groups of ICG premixing with albumin and ICG premixing with IRDye-700DX-IgG (*p* < 0.05).

To determine the optimal preloading time of ICG and albumin, the influence of fluorescence intensity of ICG as a function of premixing time was monitored at room temperature and is shown in Figure 7(b). The initial time point was chosen at 1 min after mixing, and the plot represents the binding kinetics of ICG and albumin over the time extended up to 30 min. The plot shows the change in fluorescence intensity over time of ICG interacting with albumin, demonstrating a gradual intensification of the signal. The peak fluorescence intensity was recorded at 15 min after mixing. This indicated that ICG had reached a maximum binding ratio to albumin at this time point without agitation at the room temperature. Further preloading time should be prevented as ICG exhibited an irreversible degradation profile in aqueous solution for longer time periods (results not shown).

## 4. Discussion

Sentinel lymph node status is one of the most important prognostic factors guiding adjuvant treatment in breast and other cancers, and surgical lymph node dissection, followed by histopathology, has become a standard care for determining the nodal stage of the disease [1]. However, the removal of lymph nodes, even sentinel nodes, can impact the quality of life of patients [6] as the surgery carries a significant risk of morbidity, and with the majority of lymph nodes identified as cancer negative [7], overtreatment is a serious concern. These limitations of standard lymph node biopsy have led to a demand for the development of an approach to estimate tumor burden lymph nodes noninvasively [41]. A paired-agent imaging of the sentinel lymph nodes (PAISLY) method is one approach that has recently been developed to fulfill this demand, and early preclinical work in a mouse model of human breast metastatic cancer demonstrated that PAISLY could noninvasively detect microscopic levels of tumor burden in the lymph nodes [16]. Translation of PAISLY to the clinic will require FDA approval of a targeted and a control imaging agent, yet translation could be accelerated with the use of an existing FDA-approved control imaging-agent, such as indocyanine green (ICG). The results of this work support the eventual combination of ICG with albumin (both FDA approved) with potential fluorescent-targeted antibodies (of which a number of groups are making it into clinical trials [42]). Since ICG and albumin are both FDA-approved molecules, mixing of these two molecules by a licensed pharmacist or physician is allowed under Section 503A of the Federal Food, Drug and Cosmetic Act. With no FDA-approved fluorescent antibodies to date, clinical translation will ultimately require toxicology studies of GMP fluorescent antibodies (of which there are a number of groups now using these for other applications) mixed with albumin and ICG; however, the ability to use albumin/ICG as a control offers significant advantages over having to design a new control imaging agent that would require costly GMP design, production, and manufacturing. This study experimentally demonstrated that ICG can indeed be used as a control agent in PAISLY methods that employ antibody-based targeted imaging agents, as long as the ICG is premixed with albumin prior to mixing with the antibody-based agent.

There are other available FDA-approved fluorescent imaging agents that have the potential to be used as control imaging agents, such as fluorescein or methylene blue, which have been widely used in clinical applications already [3]. ICG was chosen as it emits light in the near-infrared where light absorption in tissue and tissue autofluorescence is relatively low, allowing imaging sensitivity deeper in tissue [43]. Methylene blue also emits within the near-infrared range and would be an option; however, the main concern is that it exhibits only partial binding to proteins in lymph fluid. This lead to an observation of fast (free associated dye) and slow (protein bound dye) components of methylene blue arrival at lymph nodes and kinetics that would not be exhibited by antibody-based imaging agents [14].

For use as a control agent in PAISLY, some nonoptimal features of ICG had to be accounted for. For instance, the fluorescence quantum yield and spectrum of ICG is nonlinear versus concentration [37]. To overcome this first limitation, ICG fluorescence was measured with the Pearl System over a large range of concentrations, and a nearly 3-orders-of-magnitude linear range in concentration was identified with a high-end concentration of approximately 1 *μ*M. Since the imaging agent concentration has been found to dilute by a factor of about 10–100 from the injection site to lymph node [16], this high-end concentration was selected as the optimal concentration to inject to ensure that all signals were linear with concentration during *in vivo* imaging. Another complicating characteristic of ICG is its propensity to bind nonconvalently to proteins in the blood plasma, such as albumin, which enhances its fluorescence depending on the extent of protein binding [44]. In this study, it was demonstrated that concentration ratios of more than 50 : 1 (albumin : ICG) yielded the highest levels of ICG fluorescence, which would remain stable as a function of ICG concentration throughout lymph node imaging. The affinity of ICG to noncovalently bind to proteins added a further complication for use in PAISLY as it was found that addition of ICG to the antibody-based imaging agent changed the fluorescence of the ICG, suggesting that ICG was binding to the antibody protein. This is problematic for PAISLY, as (1) this interaction could interfere with the ability of the antibody to bind to the biomolecular target, and (2) for ICG to act as an ideal control agent, its kinetics should approximate those of the freely associated targeted agent only (but with binding to the targeted agent, it will represent both bound and free fractions of the targeted agent, at least in part). The solution to this problem was to mix the ICG with albumin at greater than a 50:1 albumin:ICG ratio (Figure 6(b)) for more than 15 min (Figure 7(b)) prior to mixing with the antibody-based agent to minimize the number of free ICG molecules able to associate with the antibody instead of the albumin. It should also be noted that, by ensuring that the vast majority of the ICG is bound to albumin prior to injection, studies have demonstrated that the ICG will drain preferentially through lymphatic vessels when injected intradermally [45]: similar to the antibody-based agents [8], which makes the albumin-bound ICG a much better candidate to act as the control imaging agent.

To test the potential for ICG to work as a control agent in PAISLY for antibody-based targeted agents, its uptake was compared against IRDye-700DX-labeled_IgG, which has already been demonstrated to be an adequate control imaging agent for an antibody-based targeted imaging agent in PAISLY [16]. The idea is that future studies could replace the IgG in the untargeted IRDye-700DX-IgG with a targeted antibody, allowing the ICG:albumin to act as a control agent in PAISLY for the IRDye-700DX-labeled targeted antibody. IRDye-700DX is an ideal fluorophore to be paired with ICG. Its emission is around 700 nm, far from the 800 nm emission peak of ICG to minimize crosstalk but still in the near-infrared window where light absorption in tissue is low. Note that a crosstalk experiment was carried out (results not shown) for 1 rat injected with ICG alone and 1 rat injected with IRDye 700DX-IgG alone. Change in the signal in the crosstalk channel was less than 0.1% of the total change in the signal in the expected channel in both cases. There is also a similarity in optical properties at 700 and 800 nm in most tissues, which means that imaging at either wavelength will interrogate the same volume/depth of tissue, allowing normalization of signal in paired-agent imaging to account for optical property, system detection, and imaging agent concentration differences between the two wavelength ranges [40]. While there are no FDA-approved IRDye-700DX-labeled targeted antibodies, good manufacturing practices- (GMP-) produced IRDye-700DX can be acquired from the manufacturer, and the dye is currently under several clinical trials having exhibited excellent safety [17, 18]. Furthermore, a GMP-produced IRDye-700DX-cetuximab (antibody for epidermal growth factor receptor) has recently been taken through toxicity studies in preparation for preliminary clinical studies [46], which could open the way to more IRDye-700DX-labeled antibodies acquiring clinical approval in the coming years.

The rat popliteal lymph node was chosen as a proof-of-principle test case for comparing the kinetics of ICG and IRDye-700DX-IgG since this node is relatively large and tends to be spatially isolated from other nodes (so there are no adjacent nodes that could obfuscate kinetic measurements in the node of interest). The area it drains is also well defined and includes the foot, footpad, and hind leg [47], which are all drained through large lymphatic vessels that also show up on wide-field fluorescent imaging (Figures 2 and 3(a)) and can be helpful for understanding the impact of the afferent vessel fluorescent agent “input functions” [14]. Considering interstitial fluid movement is intrinsically linked to lymphatic drainage, Swartz et al. demonstrated that the infusion rate of imaging agents was linearly proportional to infusion pressure in lymphatic drainage [48]. Therefore, for examining the systemic drainage mechanisms of an imaging agent, large changes in interstitial pressure upon imaging agent administration should be avoided, as it is possible that pressure-based delivery of agents to the lymph node may lead to larger discrepancies of targeted and control agent delivery rates owing to differences in size compared to delivery through physiological levels of lymph fluid flow rate (no rigorous testing was done to prove this; however, past experience has suggested high volume, and fast injections yield greater differences in paired-agent delivery rates). High tissue pressures were avoided in this study by injecting a small volume of imaging agents (0.05 mL) over a relatively long period of time (∼15 s) using a 30-gauge syringe-needle, intradermally into the right rear footpad of rats. Caution had to be taken while injecting to ensure that the needle was not too deep, as deep injections lead to delayed uptake of imaging agents into the lymphatic system [49].

After paired-agent injection, peak fluorescence signal peak was observed at 6 ± 2 min for both ICG and IRDye-700DX-IgG (Figure 3(c)). The *in vivo* results demonstrated an excellent ability for ICG to match the delivery and nonspecific retention of the antibody-based imaging agent, IRDye-700DX-IgG, using the optimal imaging agent mixing protocol described. It should be emphasized that the mixing protocol was critical as the kinetics of ICG and IRDye-700DX-IgG failed to correlate significantly with each other when ICG, IRDye-700DX-IgG, and albumin were mixed directly without preloading ICG with albumin immediately prior to injection (Figure 3(e)). With the early failure of this method, few datasets were collected, so findings were not statistically significant, but it appeared that under these condition, ICG exhibited a slower initial delivery to the lymph node (lower initial peak), while the IRDye-700DX exhibited heightened nonspecific retention (slower washout from the node). It is not clear what the causes of these differences were; however, it is possible that ICG delivery was reduced owing to binding of remaining free ICG to more stationary proteins *in vivo* upon injection; and it is possible that ICG binding to the IRDye-700DX-IgG could have led to slower washout from the lymph node. Thus, the optimal mixing protocol is required to ensure that ICG is completely bound to albumin prior to mixing with the antibody-based imaging agent.

While the presented method and results demonstrated that ICG has the potential to act as the control agent in PAISLY, for this proposed approach to be ultimately effective in replacing conventional lymph node dissection to evaluate lymph node metastasis, the targeted imaging agent must be able to identify the highest fraction of metastatic breast cancer cells. Recently, Tafreshi et al. proposed that carbonic anhydrase isoforms 9 and 12, CAIX, and CAXII, respectively, were potential targets as reliable intrinsic biomarkers for cancer targeting, as one or the other of these two markers were found to be expressed in 100% of breast cancer lymph node metastasis surveyed [10]. As a result, future studies will adapt PAISLY to dual targeting of CAIX and CAXII with ICG as a control imaging agent to maximize the sensitivity to breast cancer lymph node metastasis for all patients potentially.

## 5. Conclusion

This study indicated that ICG could fill the role of a control for the PAISLY approach to account for nonspecific uptake of an antibody imaging agent. It has demonstrated the excellent level of cancer sensitivity in rat studies to assess lymph node metastasis.

## Data Availability

The datasets generated during and/or analyzed during the current study are available from the corresponding author upon request.
